# HIV/AIDS-related non-Hodgkin’s lymphomas and confounders: preliminary report of the Sub-Saharan Africa Lymphoma Consortium (SSALC)

**DOI:** 10.1186/1750-9378-7-S1-P11

**Published:** 2012-04-19

**Authors:** Leona W Ayers, E Akin Abayomi, Clement Adebamowo, David K Chumba, Yawale Iliyasu, Kikkeri N Naresh, Joseph R NDung’u, Yvonne Perner, Wendy Stevens, Lynnette K Tumwine

**Affiliations:** 1 The Ohio State University Department of Pathology, Columbus, OH, USA; 2 Stellenbosch University Division of Haematology, Cape Town, South Africa; 3 Institute of Human Virology, Abuja, Nigeria; University of Maryland School of Medicine, Baltimore, MD, USA; 4 Moi University Department of Human Pathology and Forensic Medicine, Eldoret, Kenya; 5 Ahmadu Bello University Teaching Hospital Department of Pathology, Zaria, Nigeria; 6 Imperial College Department of Medicine, London, England, United Kingdom; 7 University of Nairobi Department of Pathology, Nairobi, Kenya; 8 University of the Witwatersrand School of Pathology, Division of Anatomical Pathology, National Health Laboratory Service, Johannesburg, South Africa; 9 University of the Witwatersrand School of Pathology, Division of Molecular Medicine and Haematology, National Health Laboratory Service, Johannesburg, South Africa; 10 Makerere University Department of Pathology, Kampala, Uganda

## Background

SSALC was established to characterize HIV/AIDS-related lymphoma and the indigenous background of malignant lymphomas (ML) in sub-Saharan Africa. Because WHO classified lymphoma subgroups can vary in prevalence African, Asian or European ancestry, we surveyed lymphoma heterogeneity in geographically diverse East, South and West sub-Saharan populations, particularly for HIV/AIDS associated immunophenotypes.

## Methods

A consortium of African pathologists, hematologist/oncologists and oncologic surgeons contributed ML cases and participated in sub-grouping according to WHO classification criteria after appropriate Institutional Review Board (IRB) approvals, Memoranda of Understanding and Material Transfer Agreements were obtained. Paraffin blocks were examined for tissue morphology (H&E), immunophenotype (34 antibodies IHC), EBER, *kappa* and *lambda* light chains (CISH) and c-myc and bcl2 translocations (FISH). HIV/AIDS diversity controls were contributed from Europe by consortium and USA by ACSR.

## Results

Consortium members contributed 46 - 368 cases each with 1408 total cases to date: 246 diffuse large B-cell lymphoma (DLBCL), 296 Burkitt lymphoma, 163 Hodgkin disease, 69 plasma cell proliferative disorders and 644 others. Aggressive DLBCL, plasmacytoma/plasmablastic lymphoma, KSHV disease and lymphoid hyperplasia will be highlighted.

## Conclusions

Sub-Saharan Africa has a variety of ML subgroups; true incidence altered by: 1) Aspiration vs. biopsy for diagnosis; 2) HIV status not communicated to pathologist; 3) known HIV/AIDS patients not biopsied; 4) initial diagnosis by morphology alone, 5) tissue preservation/processing variable.. General observations: HIV/AIDS-related lymphoma is more likely EBER+, has higher cell proliferation rates, and unfavorable immunophenotypes; regions differ in HIV clades with South (clade C) having the most “immunosuppression” associated lymphoma subgroups; East region has more pre-T lymphoblastic lymphomas and West region has more follicular lymphomas. Confounders: infectious lymphadenopathies (EBV+ lymphoproliferations), undifferentiated neuroblastomas, neuroectodermal tumors (PNETs), poorly differentiated, metastatic carcinomas and malignant melanoma (amelanotic).

